# A Very Long Foreign Body in the Bladder

**DOI:** 10.1155/2011/323197

**Published:** 2011-05-23

**Authors:** Atsushi Imai, Yuichiro Suzuki, Yasuhiro Hashimoto, Atsushi Sasaki, Hisao Saitoh, Chikara Ohyama

**Affiliations:** ^1^Kidney Research Institute, Hirosaki Hospital, 90 Yamazaki Kozawa, Hirosaki 036-8243, Japan; ^2^Department of Urology, Hirosaki University Graduate School of Medicine, 5 Zaifu-Cyo, Hirosaki, Aomori 036-8562, Japan

## Abstract

In the urinary tract, foreign body is most commonly found in the urinary bladder. But it is anatomically very difficult for a man to self-insert a long object into the urinary bladder. Here we report a case of a 49-year-old Japanese man who has inserted a 140-cm vinyl tube in the bladder for masturbation. He could not retrieve it, and the bladder foreign body remained in this position for about two years. He was referred to our hospital and open surgery was performed.

## 1. Introduction

We report here a case of a foreign body, a 140-cm vinyl tube, in the bladder. A 49-year-old Japanese man had inserted this tube into his urethra for masturbation and could not retrieve it; it remained in this position for approximately two years.

## 2. Case Summary

**Figure 1:**
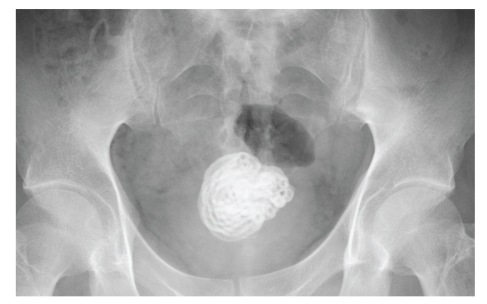
Abdominal plain X-ray showing a convoluted foreign body in the bladder.

**Figure 2:**
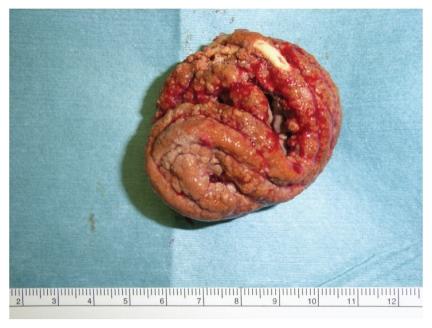
The convoluted stone-like foreign body in the bladder.

**Figure 3:**
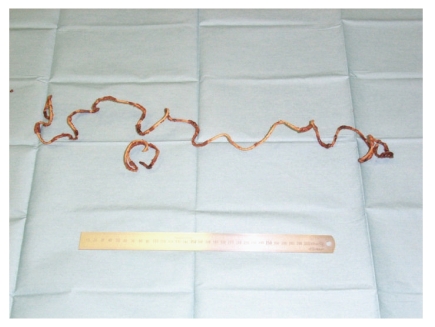
The vinyl tube was approximately 140 cm in length.

The patient was a 49-year-old male with no history of any psychiatric disorder. Gross hematuria was noted. He underwent medical examination at the local emergency room and was referred to us for examination of a foreign body in the bladder, which had been identified by ultrasonography. On physical examination, the patient did not report any abdominal pain. A plain X-ray revealed a convoluted foreign body measuring approximately 5 cm in diameter as well as a thickening in the bladder wall (Figure 1). Cystoscopy indicated a stone-like nature of the foreign body. According to the patient, approximately two years ago, he had inserted a vinyl tube into his urethra for masturbation and was unable to retrieve it; it had remained in this position, because of which he experienced urethral pain for the first six months after the incident. Because the foreign body was large, surgery by the transurethral approach was considered difficult. Therefore, suprapubic approach was performed under general anesthesia, and almost the entire foreign body mass was excised in a single lump (Figure 2). The stone-like foreign body (vinyl tube) was approximately 140 cm in length and was considered to have been intravesically curled into a spiral (Figure 3). The patient's postoperative course was uneventful.

## 3. Discussion

In the urinary tract, a foreign body is most commonly found in the urinary bladder. Self-insertion of a foreign body during masturbation has been associated with dementia and psychiatric abnormality [1].

It is anatomically very difficult for a man to self-insert a long object into the bladder. One previous case report discussed insertion of a foreign body, 95 cm in length, under similar circumstances [2], and another report discussed the case of a 12-year-old girl who inserted a 142-cm-long electrical cord into her bladder [3]. The unusual feature of the present case is that the object was left unattended for two years. In most cases, foreign bodies in the bladder are removed by cystoscopy, but in this case, an open surgery was required because of the large size of the foreign body.
